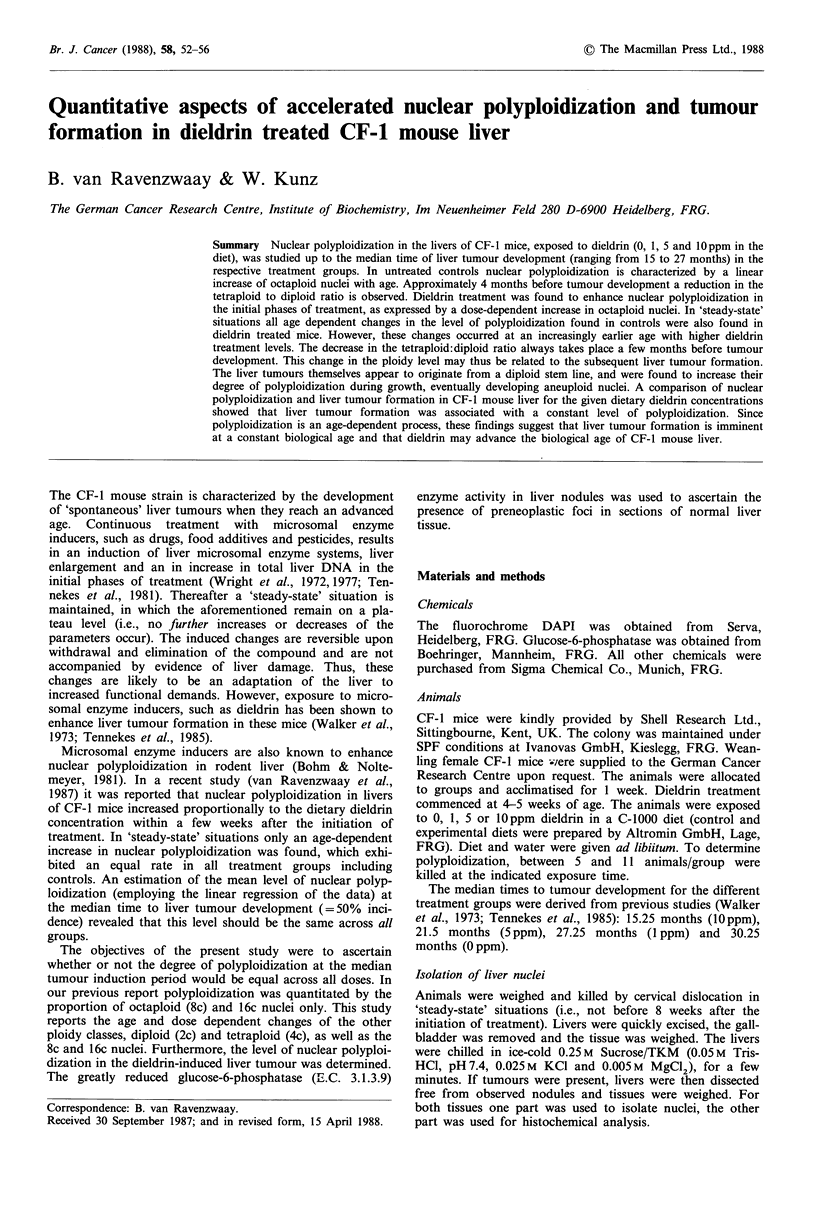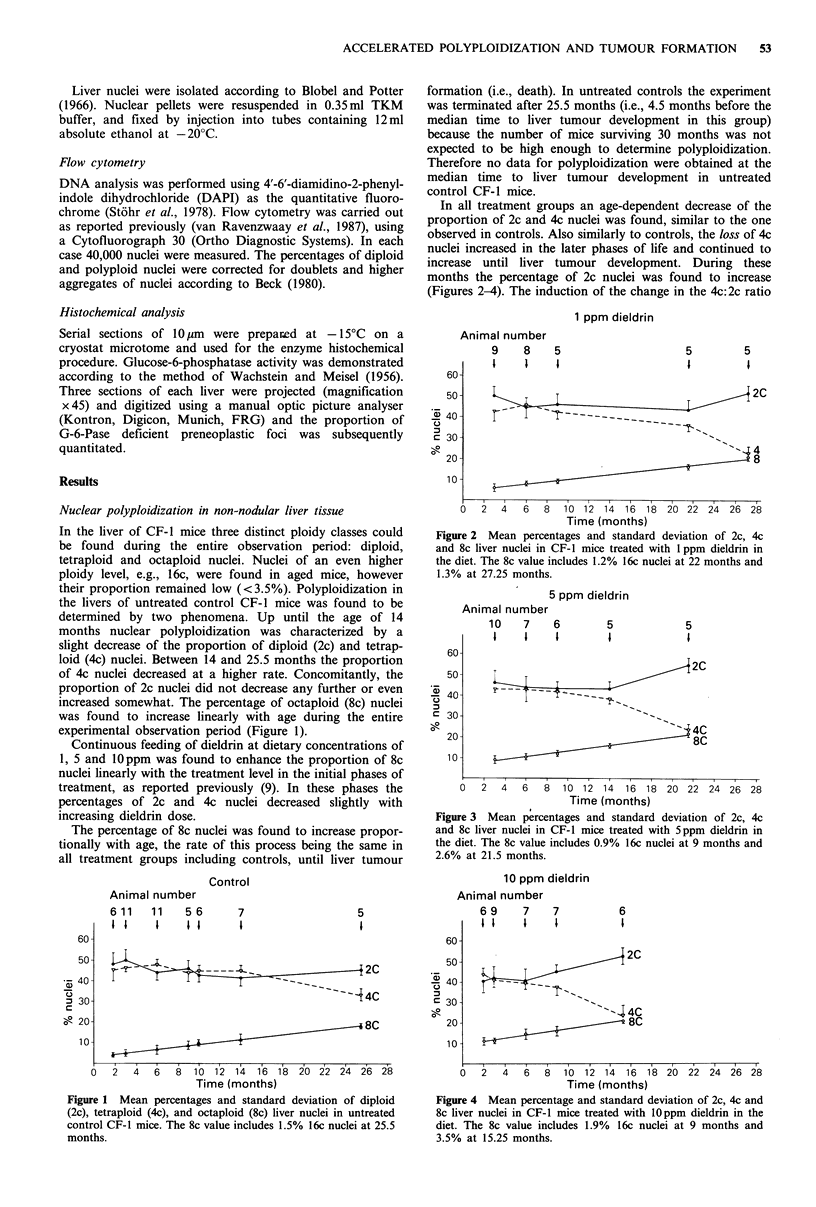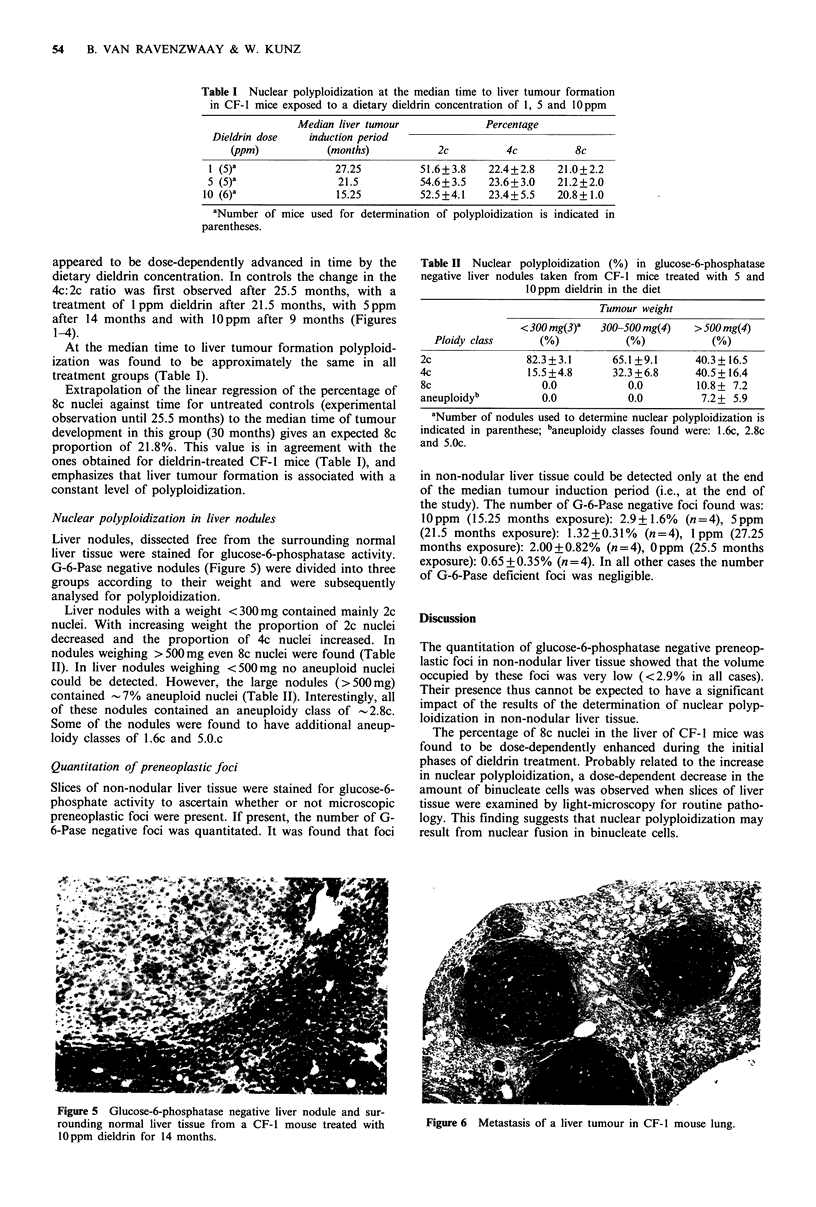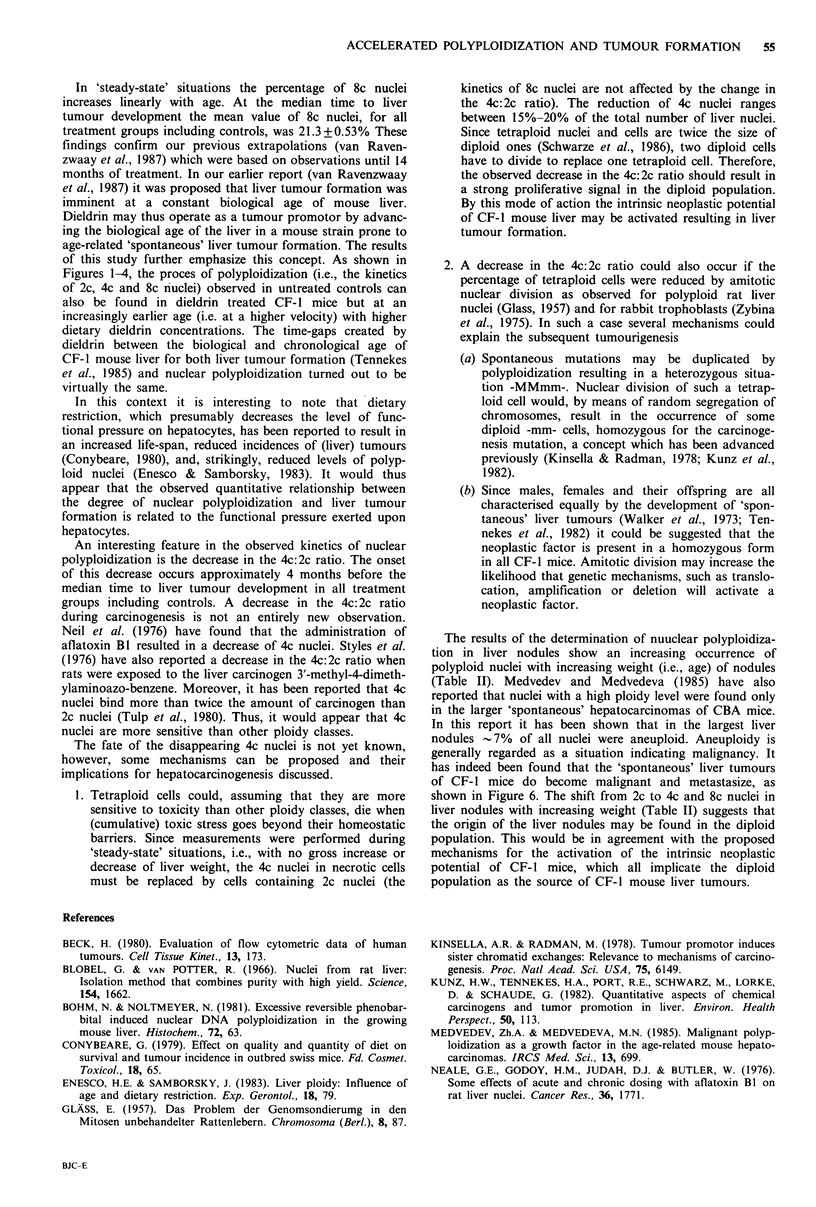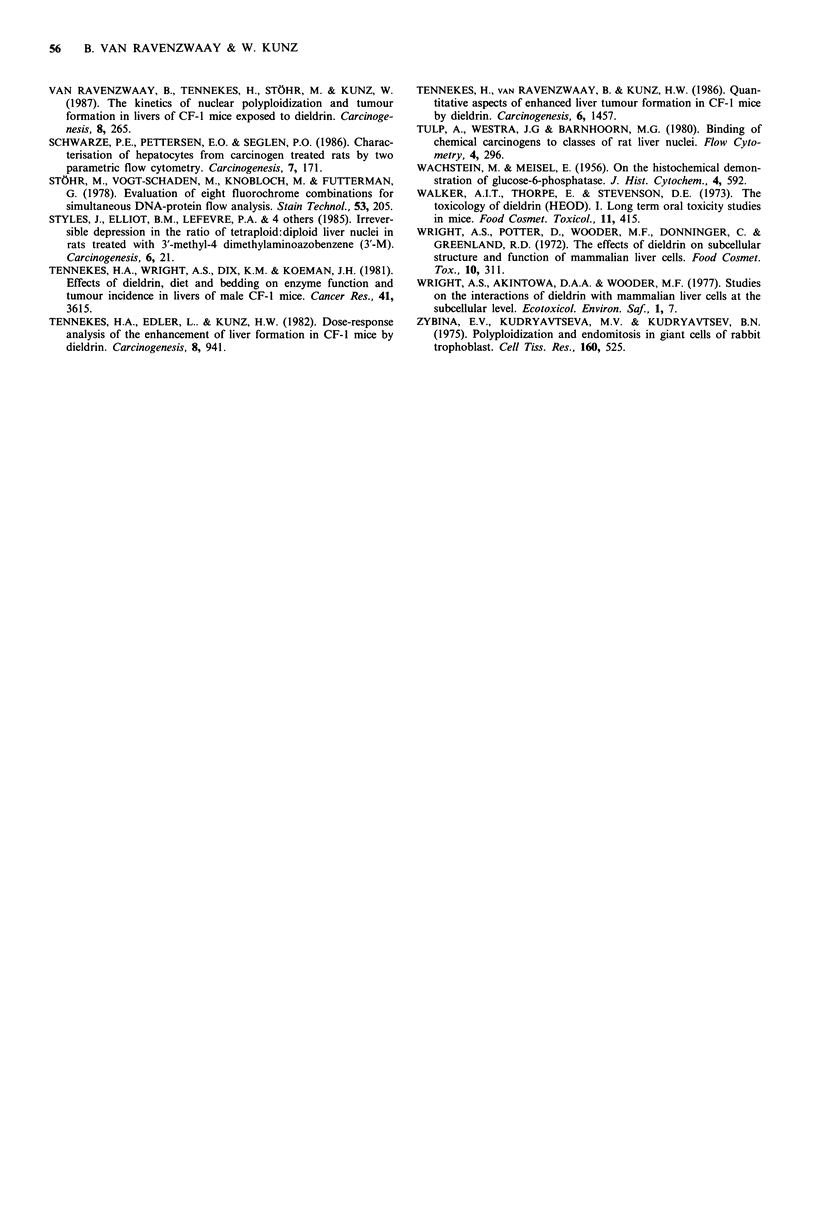# Quantitative aspects of accelerated nuclear polyploidization and tumour formation in dieldrin treated CF-1 mouse liver.

**DOI:** 10.1038/bjc.1988.160

**Published:** 1988-07

**Authors:** B. van Ravenzwaay, W. Kunz

**Affiliations:** German Cancer Research Centre, Institute of Biochemistry, Heidelberg.

## Abstract

**Images:**


Br. J. Cancer (1988), 58, 52–56 © The Macmillan Press Ltd., 1988

### Quantitative aspects of accelerated nuclear polyploidization and tumour formation in dieldrin treated CF-1 mouse liver

B. van Ravenzwaay & W. Kunz

*The German Cancer Research Centre, Institute of Biochemistry, Im Neuenheimer Feld 280 D-6900 Heidelberg, FRG.*

**Summary** Nuclear polyploidization in the livers of CF-1 mice, exposed to dieldrin (0, 1, 5 and 10 ppm in the diet), was studied up to the median time of liver tumour development (ranging from 15 to 27 months) in the respective treatment groups. In untreated controls nuclear polyploidization is characterized by a linear increase of octaploid nuclei with age. Approximately 4 months before tumour development a reduction in the tetraploid to diploid ratio is observed. Dieldrin treatment was found to enhance nuclear polyploidization in the initial phases of treatment, as expressed by a dose-dependent increase in octaploid nuclei. In 'steady-state' situations all age dependent changes in the level of polyploidization found in controls were also found in dieldrin treated mice. However, these changes occurred at an increasingly earlier age with higher dieldrin treatment levels. The decrease in the tetraploid:diploid ratio always takes place a few months before tumour development. This change in the ploidy level may thus be related to the subsequent liver tumour formation. The liver tumours themselves appear to originate from a diploid stem line, and were found to increase their degree of polyploidization during growth, eventually developing aneuploid nuclei. A comparison of nuclear polyploidization and liver tumour formation in CF-1 mouse liver for the given dietary dieldrin concentrations showed that liver tumour formation was associated with a constant level of polyploidization. Since polyploidization is an age-dependent process, these findings suggest that liver tumour formation is imminent at a constant biological age and that dieldrin may advance the biological age of CF-1 mouse liver.

The CF-1 mouse strain is characterized by the development of 'spontaneous' liver tumours when they reach an advanced age. Continuous treatment with microsomal enzyme inducers, such as drugs, food additives and pesticides, results in an induction of liver microsomal enzyme systems, liver enlargement and an in increase in total liver DNA in the initial phases of treatment (Wright *et al.*, 1972, 1977; Tennekes *et al.*, 1981). Thereafter a 'steady-state' situation is maintained, in which the aforementioned remain on a plateau level (i.e., no *further* increases or decreases of the parameters occur). The induced changes are reversible upon withdrawal and elimination of the compound and are not accompanied by evidence of liver damage. Thus, these changes are likely to be an adaptation of the liver to increased functional demands. However, exposure to microsomal enzyme inducers, such as dieldrin has been shown to enhance liver tumour formation in these mice (Walker *et al.*, 1973; Tennekes *et al.*, 1985).

Microsomal enzyme inducers are also known to enhance nuclear polyploidization in rodent liver (Bohm & Noltemeyer, 1981). In a recent study (van Ravenzwaay *et al.*, 1987) it was reported that nuclear polyploidization in livers of CF-1 mice increased proportionally to the dietary dieldrin concentration within a few weeks after the initiation of treatment. In 'steady-state' situations only an age-dependent increase in nuclear polyploidization was found, which exhibited an equal rate in all treatment groups including controls. An estimation of the mean level of nuclear polyploidization (employing the linear regression of the data) at the median time to liver tumour development (=50% incidence) revealed that this level should be the same across *all* groups.

The objectives of the present study were to ascertain whether or not the degree of polyploidization at the median tumour induction period would be equal across all doses. In our previous report polyploidization was quantitated by the proportion of octaploid (8c) and 16c nuclei only. This study reports the age and dose dependent changes of the other ploidy classes, diploid (2c) and tetraploid (4c), as well as the 8c and 16c nuclei. Furthermore, the level of nuclear polyploidization in the dieldrin-induced liver tumour was determined. The greatly reduced glucose-6-phosphatase (E.C. 3.1.3.9) enzyme activity in liver nodules was used to ascertain the presence of preneoplastic foci in sections of normal liver tissue.

Correspondence: B. van Ravenzwaay.
Received 30 September 1987; and in revised form, 15 April 1988.

### Materials and methods

### *Chemicals*

The fluorochrome DAPI was obtained from Serva, Heidelberg, FRG. Glucose-6-phosphatase was obtained from Boehringer, Mannheim, FRG. All other chemicals were purchased from Sigma Chemical Co., Munich, FRG.

### *Animals*

CF-1 mice were kindly provided by Shell Research Ltd., Sittingbourne, Kent, UK. The colony was maintained under SPF conditions at Ivanovas GmbH, Kieslegg, FRG. Weanling female CF-1 mice were supplied to the German Cancer Research Centre upon request. The animals were allocated to groups and acclimatised for 1 week. Dieldrin treatment commenced at 4–5 weeks of age. The animals were exposed to 0, 1, 5 or 10 ppm dieldrin in a C-1000 diet (control and experimental diets were prepared by Altromin GmbH, Lage, FRG). Diet and water were given *ad libiitum*. To determine polyploidization, between 5 and 11 animals/group were killed at the indicated exposure time.

The median times to tumour development for the different treatment groups were derived from previous studies (Walker *et al.*, 1973; Tennekes *et al.*, 1985): 15.25 months (10 ppm), 21.5 months (5 ppm), 27.25 months (1 ppm) and 30.25 months (0 ppm).

### *Isolation of liver nuclei*

Animals were weighed and killed by cervical dislocation in 'steady-state' situations (i.e., not before 8 weeks after the initiation of treatment). Livers were quickly excised, the gallbladder was removed and the tissue was weighed. The livers were chilled in ice-cold 0.25 M Sucrose/TKM (0.05 M Tris-HCl, pH 7.4, 0.025 M KCl and 0.005 M $MgCl_2$), for a few minutes. If tumours were present, livers were then dissected free from observed nodules and tissues were weighed. For both tissues one part was used to isolate nuclei, the other part was used for histochemical analysis.

Liver nuclei were isolated according to Blobel and Potter (1966). Nuclear pellets were resuspended in 0.35 ml TKM buffer, and fixed by injection into tubes containing 12 ml absolute ethanol at −20°C.

*Flow cytometry*

DNA analysis was performed using 4′-6′-diamidino-2-phenylindole dihydrochloride (DAPI) as the quantitative fluorochrome (Stöhr *et al.*, 1978). Flow cytometry was carried out as reported previously (van Ravenzwaay *et al.*, 1987), using a Cytofluorograph 30 (Ortho Diagnostic Systems). In each case 40,000 nuclei were measured. The percentages of diploid and polyploid nuclei were corrected for doublets and higher aggregates of nuclei according to Beck (1980).

*Histochemical analysis*

Serial sections of 10 μm were prepared at −15°C on a cryostat microtome and used for the enzyme histochemical procedure. Glucose-6-phosphatase activity was demonstrated according to the method of Wachstein and Meisel (1956). Three sections of each liver were projected (magnification ×45) and digitized using a manual optic picture analyser (Kontron, Digicon, Munich, FRG) and the proportion of G-6-Pase deficient preneoplastic foci was subsequently quantitated.

### Results

*Nuclear polyploidization in non-nodular liver tissue*

In the liver of CF-1 mice three distinct ploidy classes could be found during the entire observation period: diploid, tetraploid and octaploid nuclei. Nuclei of an even higher ploidy level, e.g., 16c, were found in aged mice, however their proportion remained low (<3.5%). Polyploidization in the livers of untreated control CF-1 mice was found to be determined by two phenomena. Up until the age of 14 months nuclear polyploidization was characterized by a slight decrease of the proportion of diploid (2c) and tetraploid (4c) nuclei. Between 14 and 25.5 months the proportion of 4c nuclei decreased at a higher rate. Concomitantly, the proportion of 2c nuclei did not decrease any further or even increased somewhat. The percentage of octaploid (8c) nuclei was found to increase linearly with age during the entire experimental observation period (Figure 1).

Continuous feeding of dieldrin at dietary concentrations of 1, 5 and 10 ppm was found to enhance the proportion of 8c nuclei linearly with the treatment level in the initial phases of treatment, as reported previously (9). In these phases the percentages of 2c and 4c nuclei decreased slightly with increasing dieldrin dose.

The percentage of 8c nuclei was found to increase proportionally with age, the rate of this process being the same in all treatment groups including controls, until liver tumour formation (i.e., death). In untreated controls the experiment was terminated after 25.5 months (i.e., 4.5 months before the median time to liver tumour development in this group) because the number of mice surviving 30 months was not expected to be high enough to determine polyploidization. Therefore no data for polyploidization were obtained at the median time to liver tumour development in untreated control CF-1 mice.

In all treatment groups an age-dependent decrease of the proportion of 2c and 4c nuclei was found, similar to the one observed in controls. Also similarly to controls, the *loss* of 4c nuclei increased in the later phases of life and continued to increase until liver tumour development. During these months the percentage of 2c nuclei was found to increase (Figures 2–4). The induction of the change in the 4c:2c ratio

**Figure 1** Mean percentages and standard deviation of diploid (2c), tetraploid (4c), and octaploid (8c) liver nuclei in untreated control CF-1 mice. The 8c value includes 1.5% 16c nuclei at 25.5 months.

**Figure 2** Mean percentages and standard deviation of 2c, 4c and 8c liver nuclei in CF-1 mice treated with 1 ppm dieldrin in the diet. The 8c value includes 1.2% 16c nuclei at 22 months and 1.3% at 27.25 months.

**Figure 3** Mean percentages and standard deviation of 2c, 4c and 8c liver nuclei in CF-1 mice treated with 5 ppm dieldrin in the diet. The 8c value includes 0.9% 16c nuclei at 9 months and 2.6% at 21.5 months.

**Figure 4** Mean percentage and standard deviation of 2c, 4c and 8c liver nuclei in CF-1 mice treated with 10 ppm dieldrin in the diet. The 8c value includes 1.9% 16c nuclei at 9 months and 3.5% at 15.25 months.

**Table I** Nuclear polyploidization at the median time to liver tumour formation in CF-1 mice exposed to a dietary dieldrin concentration of 1, 5 and 10 ppm

| *Dieldrin dose (ppm)* | *Median liver tumour induction period (months)* | *Percentage* | | |
|---|---|---|---|---|
| | | *2c* | *4c* | *8c* |
| 1 (5)[a] | 27.25 | 51.6±3.8 | 22.4±2.8 | 21.0±2.2 |
| 5 (5)[a] | 21.5 | 54.6±3.5 | 23.6±3.0 | 21.2±2.0 |
| 10 (6)[a] | 15.25 | 52.5±4.1 | 23.4±5.5 | 20.8±1.0 |

[a]Number of mice used for determination of polyploidization is indicated in parentheses.

appeared to be dose-dependently advanced in time by the dietary dieldrin concentration. In controls the change in the 4c:2c ratio was first observed after 25.5 months, with a treatment of 1 ppm dieldrin after 21.5 months, with 5 ppm after 14 months and with 10 ppm after 9 months (Figures 1–4).

At the median time to liver tumour formation polyploidization was found to be approximately the same in all treatment groups (Table I).

Extrapolation of the linear regression of the percentage of 8c nuclei against time for untreated controls (experimental observation until 25.5 months) to the median time of tumour development in this group (30 months) gives an expected 8c proportion of 21.8%. This value is in agreement with the ones obtained for dieldrin-treated CF-1 mice (Table I), and emphasizes that liver tumour formation is associated with a constant level of polyploidization.

### *Nuclear polyploidization in liver nodules*

Liver nodules, dissected free from the surrounding normal liver tissue were stained for glucose-6-phosphatase activity. G-6-Pase negative nodules (Figure 5) were divided into three groups according to their weight and were subsequently analysed for polyploidization.

Liver nodules with a weight <300 mg contained mainly 2c nuclei. With increasing weight the proportion of 2c nuclei decreased and the proportion of 4c nuclei increased. In nodules weighing >500 mg even 8c nuclei were found (Table II). In liver nodules weighing <500 mg no aneuploid nuclei could be detected. However, the large nodules (>500 mg) contained ~7% aneuploid nuclei (Table II). Interestingly, all of these nodules contained an aneuploidy class of ~2.8c. Some of the nodules were found to have additional aneuploidy classes of 1.6c and 5.0.c

### *Quantitation of preneoplastic foci*

Slices of non-nodular liver tissue were stained for glucose-6-phosphate activity to ascertain whether or not microscopic preneoplastic foci were present. If present, the number of G-6-Pase negative foci was quantitated. It was found that foci in non-nodular liver tissue could be detected only at the end of the median tumour induction period (i.e., at the end of the study). The number of G-6-Pase negative foci found was: 10 ppm (15.25 months exposure): 2.9±1.6% ($n=4$), 5 ppm (21.5 months exposure): 1.32±0.31% ($n=4$), 1 ppm (27.25 months exposure): 2.00±0.82% ($n=4$), 0 ppm (25.5 months exposure): 0.65±0.35% ($n=4$). In all other cases the number of G-6-Pase deficient foci was negligible.

**Table II** Nuclear polyploidization (%) in glucose-6-phosphatase negative liver nodules taken from CF-1 mice treated with 5 and 10 ppm dieldrin in the diet

| *Ploidy class* | *Tumour weight* <br> *<300 mg(3)*[a] (%) | *300–500 mg(4)* (%) | *>500 mg(4)* (%) |
|---|---|---|---|
| 2c | 82.3±3.1 | 65.1±9.1 | 40.3±16.5 |
| 4c | 15.5±4.8 | 32.3±6.8 | 40.5±16.4 |
| 8c | 0.0 | 0.0 | 10.8± 7.2 |
| aneuploidy[b] | 0.0 | 0.0 | 7.2± 5.9 |

[a]Number of nodules used to determine nuclear polyploidization is indicated in parenthese; [b]aneuploidy classes found were: 1.6c, 2.8c and 5.0c.

### Discussion

The quantitation of glucose-6-phosphatase negative preneoplastic foci in non-nodular liver tissue showed that the volume occupied by these foci was very low (<2.9% in all cases). Their presence thus cannot be expected to have a significant impact of the results of the determination of nuclear polyploidization in non-nodular liver tissue.

The percentage of 8c nuclei in the liver of CF-1 mice was found to be dose-dependently enhanced during the initial phases of dieldrin treatment. Probably related to the increase in nuclear polyploidization, a dose-dependent decrease in the amount of binucleate cells was observed when slices of liver tissue were examined by light-microscopy for routine pathology. This finding suggests that nuclear polyploidization may result from nuclear fusion in binucleate cells.

**Figure 5** Glucose-6-phosphatase negative liver nodule and surrounding normal liver tissue from a CF-1 mouse treated with 10 ppm dieldrin for 14 months.

**Figure 6** Metastasis of a liver tumour in CF-1 mouse lung.

In 'steady-state' situations the percentage of 8c nuclei increases linearly with age. At the median time to liver tumour development the mean value of 8c nuclei, for all treatment groups including controls, was $21.3 \pm 0.53\%$ These findings confirm our previous extrapolations (van Ravenzwaay *et al.*, 1987) which were based on observations until 14 months of treatment. In our earlier report (van Ravenzwaay *et al.*, 1987) it was proposed that liver tumour formation was imminent at a constant biological age of mouse liver. Dieldrin may thus operate as a tumour promotor by advancing the biological age of the liver in a mouse strain prone to age-related 'spontaneous' liver tumour formation. The results of this study further emphasize this concept. As shown in Figures 1–4, the proces of polyploidization (i.e., the kinetics of 2c, 4c and 8c nuclei) observed in untreated controls can also be found in dieldrin treated CF-1 mice but at an increasingly earlier age (i.e. at a higher velocity) with higher dietary dieldrin concentrations. The time-gaps created by dieldrin between the biological and chronological age of CF-1 mouse liver for both liver tumour formation (Tennekes *et al.*, 1985) and nuclear polyploidization turned out to be virtually the same.

In this context it is interesting to note that dietary restriction, which presumably decreases the level of functional pressure on hepatocytes, has been reported to result in an increased life-span, reduced incidences of (liver) tumours (Conybeare, 1980), and, strikingly, reduced levels of polyploid nuclei (Enesco & Samborsky, 1983). It would thus appear that the observed quantitative relationship between the degree of nuclear polyploidization and liver tumour formation is related to the functional pressure exerted upon hepatocytes.

An interesting feature in the observed kinetics of nuclear polyploidization is the decrease in the 4c:2c ratio. The onset of this decrease occurs approximately 4 months before the median time to liver tumour development in all treatment groups including controls. A decrease in the 4c:2c ratio during carcinogenesis is not an entirely new observation. Neil *et al.* (1976) have found that the administration of aflatoxin B1 resulted in a decrease of 4c nuclei. Styles *et al.* (1976) have also reported a decrease in the 4c:2c ratio when rats were exposed to the liver carcinogen 3′-methyl-4-dimethylaminoazo-benzene. Moreover, it has been reported that 4c nuclei bind more than twice the amount of carcinogen than 2c nuclei (Tulp *et al.*, 1980). Thus, it would appear that 4c nuclei are more sensitive than other ploidy classes.

The fate of the disappearing 4c nuclei is not yet known, however, some mechanisms can be proposed and their implications for hepatocarcinogenesis discussed.

1. Tetraploid cells could, assuming that they are more sensitive to toxicity than other ploidy classes, die when (cumulative) toxic stress goes beyond their homeostatic barriers. Since measurements were performed during 'steady-state' situations, i.e., with no gross increase or decrease of liver weight, the 4c nuclei in necrotic cells must be replaced by cells containing 2c nuclei (the kinetics of 8c nuclei are not affected by the change in the 4c:2c ratio). The reduction of 4c nuclei ranges between 15%–20% of the total number of liver nuclei. Since tetraploid nuclei and cells are twice the size of diploid ones (Schwarze *et al.*, 1986), two diploid cells have to divide to replace one tetraploid cell. Therefore, the observed decrease in the 4c:2c ratio should result in a strong proliferative signal in the diploid population. By this mode of action the intrinsic neoplastic potential of CF-1 mouse liver may be activated resulting in liver tumour formation.

2. A decrease in the 4c:2c ratio could also occur if the percentage of tetraploid cells were reduced by amitotic nuclear division as observed for polyploid rat liver nuclei (Glass, 1957) and for rabbit trophoblasts (Zybina *et al.*, 1975). In such a case several mechanisms could explain the subsequent tumourigenesis
   (*a*) Spontaneous mutations may be duplicated by polyploidization resulting in a heterozygous situation -MMmm-. Nuclear division of such a tetraploid cell would, by means of random segregation of chromosomes, result in the occurrence of some diploid -mm- cells, homozygous for the carcinogenesis mutation, a concept which has been advanced previously (Kinsella & Radman, 1978; Kunz *et al.*, 1982).
   (*b*) Since males, females and their offspring are all characterised equally by the development of 'spontaneous' liver tumours (Walker *et al.*, 1973; Tennekes *et al.*, 1982) it could be suggested that the neoplastic factor is present in a homozygous form in all CF-1 mice. Amitotic division may increase the likelihood that genetic mechanisms, such as translocation, amplification or deletion will activate a neoplastic factor.

The results of the determination of nuuclear polyploidization in liver nodules show an increasing occurrence of polyploid nuclei with increasing weight (i.e., age) of nodules (Table II). Medvedev and Medvedeva (1985) have also reported that nuclei with a high ploidy level were found only in the larger 'spontaneous' hepatocarcinomas of CBA mice. In this report it has been shown that in the largest liver nodules $\sim 7\%$ of all nuclei were aneuploid. Aneuploidy is generally regarded as a situation indicating malignancy. It has indeed been found that the 'spontaneous' liver tumours of CF-1 mice do become malignant and metastasize, as shown in Figure 6. The shift from 2c to 4c and 8c nuclei in liver nodules with increasing weight (Table II) suggests that the origin of the liver nodules may be found in the diploid population. This would be in agreement with the proposed mechanisms for the activation of the intrinsic neoplastic potential of CF-1 mice, which all implicate the diploid population as the source of CF-1 mouse liver tumours.

### References

BECK, H. (1980). Evaluation of flow cytometric data of human tumours. *Cell Tissue Kinet.*, **13**, 173.

BLOBEL, G. & VAN POTTER, R. (1966). Nuclei from rat liver: Isolation method that combines purity with high yield. *Science*, **154**, 1662.

BOHM, N. & NOLTMEYER, N. (1981). Excessive reversible phenobarbital induced nuclear DNA polyploidization in the growing mouse liver. *Histochem.*, **72**, 63.

CONYBEARE, G. (1979). Effect on quality and quantity of diet on survival and tumour incidence in outbred swiss mice. *Fd. Cosmet. Toxicol.*, **18**, 65.

ENESCO, H.E. & SAMBORSKY, J. (1983). Liver ploidy: Influence of age and dietary restriction. *Exp. Gerontol.*, **18**, 79.

GLÄSS, E. (1957). Das Problem der Genomsondierumg in den Mitosen unbehandelter Rattenlebern. *Chromosoma* (*Berl.*), **8**, 87.

KINSELLA, A.R. & RADMAN, M. (1978). Tumour promotor induces sister chromatid exchanges: Relevance to mechanisms of carcinogenesis. *Proc. Natl Acad. Sci. USA*, **75**, 6149.

KUNZ, H.W., TENNEKES, H.A., PORT, R.E., SCHWARZ, M., LORKE, D. & SCHAUDE, G. (1982). Quantitative aspects of chemical carcinogens and tumor promotion in liver. *Environ. Health Perspect.*, **50**, 113.

MEDVEDEV, ZH.A. & MEDVEDEVA, M.N. (1985). Malignant polyploidization as a growth factor in the age-related mouse hepatocarcinomas. *IRCS Med. Sci.*, **13**, 699.

NEALE, G.E., GODOY, H.M., JUDAH, D.J. & BUTLER, W. (1976). Some effects of acute and chronic dosing with aflatoxin B1 on rat liver nuclei. *Cancer Res.*, **36**, 1771.

VAN RAVENZWAAY, B., TENNEKES, H., STÖHR, M. & KUNZ, W. (1987). The kinetics of nuclear polyploidization and tumour formation in livers of CF-1 mice exposed to dieldrin. *Carcinogenesis*, **8**, 265.

SCHWARZE, P.E., PETTERSEN, E.O. & SEGLEN, P.O. (1986). Characterisation of hepatocytes from carcinogen treated rats by two parametric flow cytometry. *Carcinogenesis*, **7**, 171.

STÖHR, M., VOGT-SCHADEN, M., KNOBLOCH, M. & FUTTERMAN, G. (1978). Evaluation of eight fluorochrome combinations for simultaneous DNA-protein flow analysis. *Stain Technol.*, **53**, 205.

STYLES, J., ELLIOT, B.M., LEFEVRE, P.A. & 4 others (1985). Irreversible depression in the ratio of tetraploid:diploid liver nuclei in rats treated with 3′-methyl-4 dimethylaminoazobenzene (3′-M). *Carcinogenesis*, **6**, 21.

TENNEKES, H.A., WRIGHT, A.S., DIX, K.M. & KOEMAN, J.H. (1981). Effects of dieldrin, diet and bedding on enzyme function and tumour incidence in livers of male CF-1 mice. *Cancer Res.*, **41**, 3615.

TENNEKES, H.A., EDLER, L.. & KUNZ, H.W. (1982). Dose-response analysis of the enhancement of liver formation in CF-1 mice by dieldrin. *Carcinogenesis*, **8**, 941.

TENNEKES, H., VAN RAVENZWAAY, B. & KUNZ, H.W. (1986). Quantitative aspects of enhanced liver tumour formation in CF-1 mice by dieldrin. *Carcinogenesis*, **6**, 1457.

TULP, A., WESTRA, J.G & BARNHOORN, M.G. (1980). Binding of chemical carcinogens to classes of rat liver nuclei. *Flow Cytometry*, **4**, 296.

WACHSTEIN, M. & MEISEL, E. (1956). On the histochemical demonstration of glucose-6-phosphatase. *J. Hist. Cytochem.*, **4**, 592.

WALKER, A.I.T., THORPE, E. & STEVENSON, D.E. (1973). The toxicology of dieldrin (HEOD). I. Long term oral toxicity studies in mice. *Food Cosmet. Toxicol.*, **11**, 415.

WRIGHT, A.S., POTTER, D., WOODER, M.F., DONNINGER, C. & GREENLAND, R.D. (1972). The effects of dieldrin on subcellular structure and function of mammalian liver cells. *Food Cosmet. Tox.*, **10**, 311.

WRIGHT, A.S., AKINTOWA, D.A.A. & WOODER, M.F. (1977). Studies on the interactions of dieldrin with mammalian liver cells at the subcellular level. *Ecotoxicol. Environ. Saf.*, **1**, 7.

ZYBINA, E.V., KUDRYAVTSEVA, M.V. & KUDRYAVTSEV, B.N. (1975). Polyploidization and endomitosis in giant cells of rabbit trophoblast. *Cell Tiss. Res.*, **160**, 525.